# Diagnostic value of routine chest tube tip culture in surgery for noninfectious lung disease

**DOI:** 10.1186/s13019-021-01713-6

**Published:** 2021-11-10

**Authors:** Martijn van Schelt, Kevin Jenniskens, Rob J. Rentenaar, Inez Bronsveld

**Affiliations:** 1 Department of Pulmonology, University Medical Center Utrecht, Utrecht, The Netherlands; 2 Julius Center for Health Sciences and Primary Care, University Medical Center Utrecht, Utrecht University, Utrecht, The Netherlands; 3 Department of Medical Microbiology, University Medical Center Utrecht, Utrecht, The Netherlands

**Keywords:** Bacterial culture, Chest tube, Infection, Lung surgery, Perioperative care, Diagnostic accuracy

## Abstract

**Background:**

Evaluation of the diagnostic value of routine chest tube tip culture for detection of postoperative infection after surgery for noninfectious lung disease.

**Methods:**

Included subjects were patients who underwent lung surgery between January 1st 2013 and January 1st 2018 in University Medical Centre Utrecht and of whom a chest tube tip was cultured. Postoperative outcomes included pneumonia, surgical site infection, and empyema within 30 days after surgery. Univariable analysis for diagnostic accuracy of chest tube tip culture results predicting these postoperative outcomes was performed, as well as multivariable analysis using penalized firth logistic regression.

**Results:**

Patients developed one or more postoperative infections in 42 out of 210 (20%) lung surgeries. Pneumonia, surgical site infection, and empyema were found in 36 (17%), 8 (4%), and 2 (1%) cases respectively. Chest tube tip culture had a sensitivity of 31%, a specificity of 83%, a positive predictive value of 32%, and a negative predictive value of 83% for postoperative infections. In the subgroup of patients who did not have evidence of postoperative infection at the time of chest tube removal, the drain tip culture’s positive and negative predictive value changed to 18% and 92% respectively. Adding additional variables to chest tube tip culture in a prediction model resulting in only limited improvement in diagnostic performance.

**Conclusions:**

We found insufficient diagnostic performance to support the practice of routine chest tube tip culture after surgery for noninfectious lung disease. Therefore, routine chest tube tip culture is not advisable and should be omitted to unburden the healthcare process and prevent low value care together with extra costs.

## Background

Despite the implementation of minimally invasive techniques like video-assisted thoracoscopic surgery (VATS) in lung surgery, postoperative pulmonary complications are frequently observed. Incidence of pulmonary complications ranges from 7 to 27%, depending on the type of procedure and patient characteristics [1–3]. Among postoperative complications after lung surgery, infectious complications are common; reported rates range from 3 to 7% for pneumonia [2, 4–6], 0.9 to 2% for empyema, [5–7] and 2 to 3% for surgical site infection (SSI) [5, 6]. Postoperative infections result in prolonged hospital stay, higher healthcare costs, and are associated with higher mortality [8]. Early detection and pathogen specific treatment are desirable for postoperative infections.

Chest tubes are placed in the pleural space after thoracic surgery to prevent development of pneumothoraces and to monitor air leak and hemothoraces. An association between chest tube tip cultures and SSI has been found in spinal surgery [9, 10]. In hip and knee arthroplasty conflicting results regarding diagnostic value of routine drain cultures are reported [11–13]. To our knowledge, only one study described the relationship between chest tube tip culture and postoperative infection in thoracic surgery. Yamauchi et al. found a positive predictive value of 41.3% for chest tube tip culture and postoperative infection in patients who underwent lung cancer surgery by thoracotomy [14].

At the University Medical Center Utrecht (UMCU), The Netherlands, chest tube tip culture is routinely performed after lung surgery to diagnose postoperative infections at an early stage. However, the diagnostic performance of this practice remains unclear. Therefore, the aim of this study was to evaluate the diagnostic value of routine chest tube tip culture for early detection of postoperative infection after surgery for noninfectious lung disease.

## Methods

### Setting

A retrospective cohort study was conducted at the UMCU, a university hospital with around 150 surgeries per year for various pulmonary diseases. In November 2018 the Utrecht Medical Research Ethics Committee confirmed that the Medical Research Involving Human Subjects Act (WMO) does not apply to this study (reference number WAG/mb/18/041978), consequently waiving the need for informed consent.

### Patients

All patients who underwent lung surgery for noninfectious disease between January 1st 2013 and January 1st 2018 of whom a chest tube tip was cultured were eligible for this study. Patients were identified retrospectively using hospital records. Exclusion criteria were hospital admission for lung transplantation surgery, and use of different perioperative antibiotic prophylaxes. Patients with a history of lung transplantation surgery were eligible for inclusion in this study.

### Procedures

Surgery was performed under general anesthesia. Perioperative antibiotics were administered in all lung surgeries. Antibiotic prophylaxis in the period between 2013 and 2015 consisted of six gifts of 1200 mg amoxicillin and clavulanic acid administered intravenously in the first 48 h postoperatively. Between 2015 and 2018 this changed to one gift of 1200 mg amoxicillin and clavulanic acid intravenously before starting surgery, and three additional gifts within the first 24 h. The duration of antibiotic prophylaxis was reduced because of lack of benefit in literature of extended use and uniform practice with other types of surgery. A chest tube was inserted in the pleural space before chest closure.

The criteria for chest tube removal were according to Dutch guidelines and varied per type of operation [15]. General criteria for chest tube removal were sufficient expansion of the lung with no or minimal pneumothorax on chest X-ray, no observation of hemothorax or chylothorax, minimal pleural fluid, drain production below 60 ml/3 h, and no signs of persistent air leakage.

The chest tube removal was done under semi-sterile circumstances. The patient was positioned on his side. The physician put on non-sterile gloves. Next, the skin and part of the chest tube was wiped with sterile gausses draped in an antiseptic as chlorhexidine 0.5% in alcohol 70%. The suture was prepared with a sterile stitch cutter. The chest tube was removed, and the tip was cut with sterile scissors.

The chest tube tip was transported in a sterile container to the microbiology laboratory. Processing was done using the roll plate method modified from Maki et al. [16]. The tip was rolled over soy agar with 5% sheep blood and chocolate agar. Plates were examined after 18 and 48 h of incubation at 35–37 °C in 5% CO_2_. Macroscopic semi quantitative assessments of growth of colonies on agar plates were performed.

### Definitions

A chest tube tip culture was found positive if any organism was cultured from a thoracic drain placed during surgery. Patients in whom more than one drain was placed during surgery were found positive if one or more drains resulted in a positive chest tube tip culture.

Postoperative infection was defined as an empyema, pneumonia or SSI occurring within 30 days after surgery. Empyema was defined as purulent effusion in pleural cavity, pleural fluid pH < 7.20 or a positive pleural fluid culture. A SSI was defined as diagnosis of SSI by the attending physician or a positive wound culture from the surgical site. Postoperative pneumonia was diagnosed using modified criteria described by Russotto et al. [17]: initiation of antibiotics for a suspected respiratory tract infection and at least one of the following criteria: new or changed sputum production; a positive sputum culture; new or changed lung opacities on a chest radiograph; temperature > 38.3 °C.

### Data collection

The following data were collected from the medical records of patients with a follow-up of 30 days postoperative: gender, age, body mass index (BMI), smoking status (current smoker, former smoker or no history of smoking), chronic obstructive pulmonary disease, diabetes mellitus, cystic fibrosis, lung transplantation in history, duration of postoperative chest drainage, date of surgery, type of surgery, surgical method, chest tube tip cultures, and cultures from pleural fluid, wound swabs, sputum and blood. Surgical methods were thoracotomy or VATS. Surgical procedures were divided in oncological ((bi)lobectomy and lymph node dissection, sleeve resection and lymph node dissection, wedge resection, pleural or mediastinal mass biopsy/excision and other procedures) and non-oncological ((bi)lobectomy, wedge resection, pleurodesis, decortication and, other procedures).

### Statistical analysis

Difference in baseline characteristics of patients with and without postoperative infections were statistically assessed using Chi-square and Fisher exact test for categorical variables, and for continuous variables Student’s t-test and Mann–Whitney U test for respectively normally and skewed distributions.

The diagnostic accuracy of chest tube tip cultures was assessed both as a single test (univariable) and in a multivariable prediction model. In the univariable analysis diagnostic accuracy of chest tube tip cultures and postoperative infections was assessed using sensitivity, specificity, positive predictive value (PPV) and negative predictive value (NPV). Firth logistic regression was used to construct a multivariable model. Five candidate predictors were included in the full model based on clinical relevance. This number of predictors was based on the method by van Smeden et al. [18], using a square root of the mean squared prediction error of 0.07. Predictor selection for the final model was based on change of the AIC compared to the full model. Chest tube tip cultures were kept in the final model regardless, as the goal is to assess the added value of other predictors to this diagnostic test. Area under the receiver operator characteristic (AUROC) curve and a calibration plot were used to assess discrimination and calibration respectively. Data were analyzed using version 25 of the IBM SPSS software [19] and R statistical software package version 3.5.3 [20]. *P* values < 0.05 were considered statistically significant.

## Results

### Study population

From January 1st 2013 to January 1st 2018, 302 lung surgeries for noninfectious disease were performed in the UMCU. 92 surgeries were excluded as no chest tube tip culture was available, leaving 210 surgeries in 202 patients available for analysis.

The median age of patients at time of surgery was 63 [IQR 51–69] years with a median BMI of 25 kg/m^2^ [IQR 22–27]. Chronic obstructive pulmonary disease and diabetes mellitus were present in 23.3% and 10.5% of patients, respectively. VATS approach was used in 59.0% of cases, a thoracotomy in 41.0%. The most common oncological operation was (bi)lobectomy (53.3%), followed by wedge resection (12.4%). In non-oncological surgery pleurodesis was the most frequently performed operation (13.8%). The median duration of postoperative chest drainage was 4 days [IQR 3–6]. Chest tube tip cultures were found positive in 19.5% of all surgical cases.

Univariable analysis showed smoking status was significantly correlated with postoperative infection status (*P* = 0.026) (Table 1). Furthermore, patients with postoperative infection more frequently had a history of lung transplantation (9.5% vs. 1.2%, *P* = 0.016), a longer duration of postoperative chest drainage (median 4 days [IQR 4–8] vs. median 4 days [IQR 3–6], *P* = 0.007) and positive chest tube culture (31% vs. 16.7%, *P* = 0.037).

**Table 1:** Patient characteristics and univariable analysis of risk factors for postoperative infection

Characteristics	Patients with postoperative infection (n = 42)	Patients without postoperative infection (n = 168)	*P* value
*Demographics*			
Age (years)	62 [53–70]	63 [50–69]	0.67
Male sex	27 (64.3)	104 (61.9)	0.78
BMI (kg/m^2^)	25 [23–29]	25 [22–27]	0.10
*Comorbidities*			
COPD	13 (31.0)	36 (21.4)	0.19
Diabetes mellitus	7 (16.7)	15 (8.9)	0.16
Lung transplantation in history	4 (9.5)	2 (1.2)	0.016
Cystic fibrosis	0 (0.0)	2 (1.2)	1.00
*Smoking status*			0.026
Never smoker	9 (21.4)	35 (20.8)	
Former smoker	31 (73.8)	95 (56.5)	
Current smoker	2 (4.8)	38 (22.6)	
*Surgical method*			0.092
VATS	20 (52.4)	104 (61.9)	
Thoracotomy	22 (47.6)	64 (38.1)	
*Surgical procedure*			0.460
*Oncological*			
(Bi)lobectomy and lymph node dissection	23 (54.8)	89 (53.0)	
Sleeve resection and lymph node dissection	4 (9.5)	6 (3.6)	
Wedge resection	2 (4.0)	24 (14.3)	
Pleural or mediastinal mass biopsy/excision	0 (0)	7 (4.2)	
Other	1 (2.4)	5 (3.0)	
*Non-oncological*			
(Bi)lobectomy	1 (2.4)	4 (2.4)	
Wedge resection	2 (4.8)	4 (2.4)	
Pleurodesis	6 (14.3)	23 (13.7)	
Decortication	2 (4.9)	3 (1.8)	
Other	1 (2.4)	3 (1.8)	
*Chest tube drainage*			
Duration of postoperative chest drainage (days)	4 [4–8]	4 [3–6]	0.007
Positive chest tube tip culture	13 (31.0)	28 (16.7)	0.037

Data are given as number n (%) or median [IQR]

BMI, Body mass index; COPD, chronic obstructive pulmonary disease; IQR, interquartile range; NS, not significant; VATS, video-assisted thoracic surgery

### Postoperative infections and chest tube tip cultures

Overall, 42/210 patients developed one or more postoperative infection. Pneumonia, SSI and empyema developed in 36 (17%), 8 (4%), and 2 (1%) patients, respectively. Pneumonia became apparent at a median of 3 days [IQR 2–7] and SSI at a median of 11 [IQR 7–13] days after surgery. The two cases of empyema occurred at day 8 and 9 postoperatively.

The relationship between chest tube tip cultures and postoperative infections is shown in Table 2. Chest tube tip cultures were positive in 13/42 patients who developed a postoperative infection, and negative in 140/168 who did not develop a postoperative infection. This resulted in a sensitivity of 31%, a specificity of 83%, a PPV of 32%, and a NPV of 83% for postoperative infections. A positive chest tube tip culture in patients with SSI, pneumonia and empyema was found in 6/8, 9/36 and 2/2 patients, respectively.

**Table 2:** Results for chest tube tip cultures and postoperative infections

Chest tube tip cultures	Postoperative infection (n = 42)	No postoperative infection (n = 168)	Postoperative infections^a^		
			SSI (n = 8)	Pneumonia (n = 36)	Empyema (n = 2)
Positive	13	28	6	9	2
Negative	29	140	2	27	0

The table results in the following estimates of accuracy for prediction of postoperative infections using a chest tube tip culture: sensitivity 31%, specificity 83%, positive predictive value 32% and negative predictive value 83%

SSI, Surgical site infection

^a^In 2 patients 2 types of infections developed and in 1 patient 3 types of infections developed

Table 3 shows patients who developed a postoperative infection *after* chest tube removal. Positive cultures were found in 6/18 cultures patients with a postoperative infection after chest tube removal. Of the 166 patients who did not develop an infection after chest tube removal, 139 of the chest tube tip cultures were negative. The chest tube tip cultures sensitivity, specificity, PPV and NPV were 33%, 84%, 18% and 92%, respectively for infection after chest tube removal. In the patients with SSI occurring after chest tube removal 3/4 chest tube tips were positive and in pneumonia occurring after chest tube removal this was the case for 3/14. No empyema developed after chest tube removal.

**Table 3:** Results for chest tube tip cultures and postoperative infections after chest tube removal

Chest tube tip cultures	Postoperative infection (n = 18)	No postoperative infection (n = 166)	Postoperative infections		
			SSI (n = 4)	Pneumonia (n = 14)	Empyema (n = 0)
Positive	6	27	3	3	0
Negative	12	139	1	11	0

The table results in the following estimates of accuracy for prediction of postoperative infections occurring after chest tube removal using a chest tube tip culture: sensitivity 33%, specificity 84%, positive predictive value 18% and negative predictive value 92%

SSI, Surgical site infection

The organisms isolated from the chest tube tip cultures are listed in Table 4. A total of 48 bacterial isolates were identified in 41 patients. Coagulase-negative staphylococci (CNS) were isolated from 32 patients with a positive culture (76%), of which 9 (28%) developed a postoperative infection. In 4/5 patients with a chest tip culture positive for *Staphylococcus aureus* (12%) a postoperative infection developed. In these 4 patients, 3 cases of SSI, 2 of pneumonia and 2 of empyema were present. All patients with a chest tube tip culture positive for *Corynebacterium* species (10%) did not develop a postoperative infection.

**Table 4:** Isolated organism from chest tube tip cultures and postoperative infections

Bacteria	Positive chest tube tip cultures^a^	Postoperative infection	Type of postoperative infection		
			SSI	Pneumonia	Empyema
Coagulase-negative staphylococci	32	9	2	7	0
*Staphylococcus aureus*^*b*^	5	4	3	2	2
Corynebacterium species	4	0	0	0	0
*Enterococcus faecalis*	3	2	1	1	0
*Enterococcus faecium*	1	1	0	1	0
*Streptococcus mitis* group	1	0	0	0	0
*Klebsiella pneumoniae*	1	1	1	0	0
*Enterobacter cloacae* complex	1	0	0	0	0

^a^In the chest tube tip culture(s) of 5 patients 2 pathogens were isolated and in 1 patient 3 pathogens were isolated

^b^A total of 7different infections developed in 4 patients with a chest tube tip culture positive for *Staphylococcus aureus*

SSI, Surgical site infection

The concordance between organisms cultured from the chest tube tip and from other specimens from the same patient, among patients with both a positive chest tube tip culture and a postoperative infectious complication is shown in Table 5. In 5/6 patients with a SSI the same organism was found in the chest tube tip culture and wound culture *S. aureus* in 4 chest tube tip/wound culture combinations and *Klebsiella pneumoniae* in one. The 2 patients with a postoperative empyema had a chest tube tip culture positive for *S. aureus*. In both patients, *S. aureus* was also found in the pleural fluid. Pneumonia developed in 9 patients with a positive chest tip culture. In none of these cases the isolates cultured from sputum matched with the organism found in the chest tube tip culture from the same patient.

**Table 5:** Concordance between cultures in patients with positive chest tube tip culture and a postoperative infection

Patient	Infection	Organism from	
		Chest tube tip	Wound, sputum, pleural effusion or blood
1	SSI	*Klebsiella pneumoniae*	Wound: *Klebsiella pneumoniae*
2	SSI, pneumonia	*Staphylococcus aureus*	Wound: *Staphylococcus aureus*
3	SSI	CNS, *Staphylococcus aureus*, *Enterococcus faecalis*	No pathogen isolated
4	SSI	CNS	Wound: CNS
5	SSI, Pneumonia, Empyema	*Staphylococcus aureus*	Wound: *Staphylococcus aureus*, pleural effusion: *Staphylococcus aureus*
6	SSI, Empyema	*Staphylococcus aureus*	Wound: *Staphylococcus aureus*, pleural effusion: *Staphylococcus aureus*
7	Pneumonia	CNS	Sputum: *Candida* species^a^
8	Pneumonia	CNS, *Enterococcus faecium*	Sputum: *Aspergillus fumigatus*, *Candida* species^a^
9	Pneumonia	CNS	No pathogen isolated
10	Pneumonia	CNS	Sputum: *Serratia* species
11	Pneumonia	CNS	No pathogen isolated
12	Pneumonia	CNS	Sputum: *Enterobacter cloacae* complex, *Klebsiella pneumoniae*
13	Pneumonia	CNS, *Enterococcus faecalis*	Sputum: *Candida* species^a^

^a^Organism not deemed causative in the clinical infection

CNS, Coagulase-negative staphylococci; SSI, surgical site infection

**Fig. 1:**
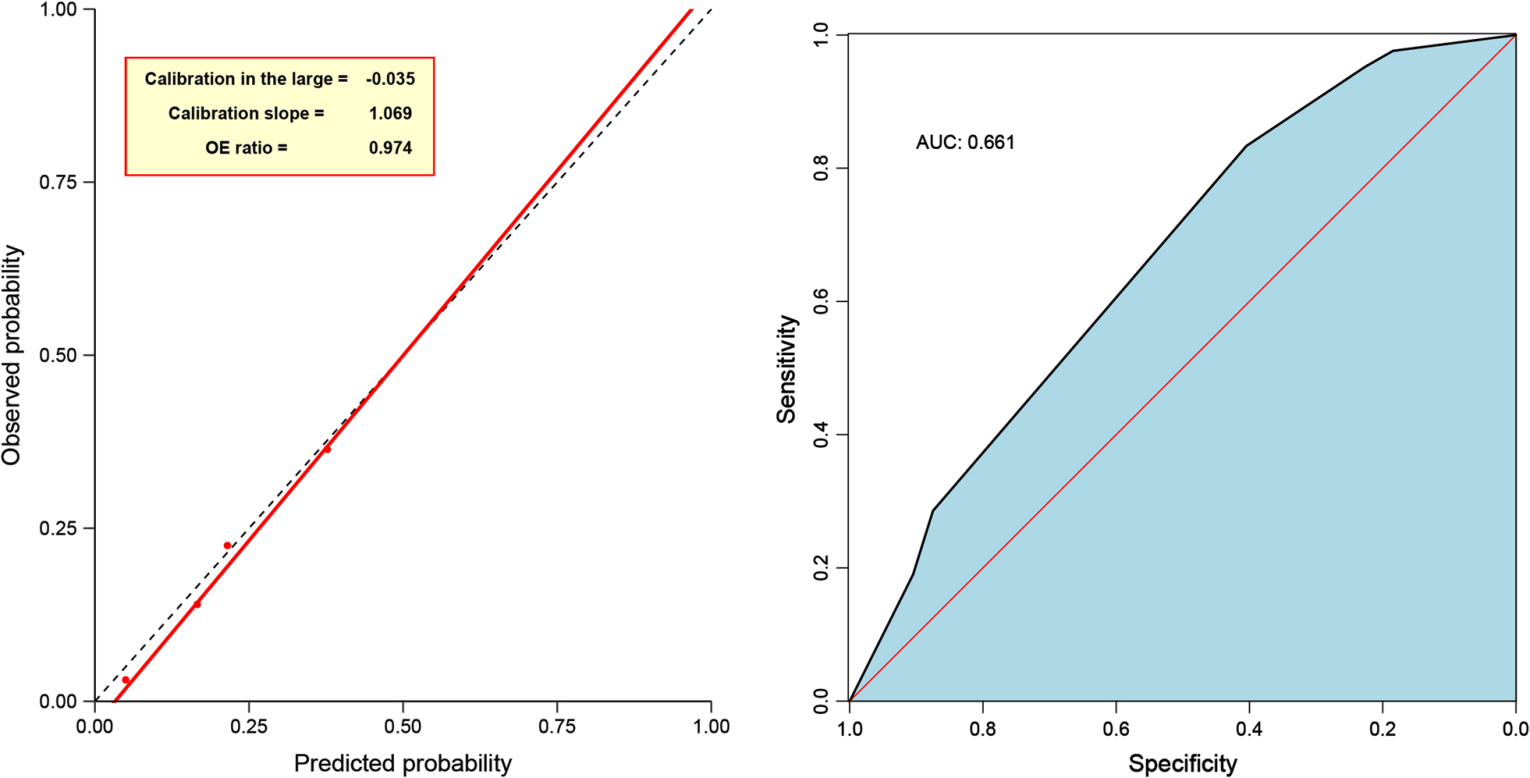
Reciever operator characteristic curve and calibration plot, with area under the curve and calibration in the large, calibration slope, and observed expected ratio

In the multivariable approach, smoking status, diabetes mellitus, duration of postoperative drainage, and type of surgery were included as predictors in the full model alongside chest tube tip culture. After predictor selection, smoking status, duration of postoperative drainage, and chest tube tips were kept as variables in the final model (Table 6). Figure 1 shows the AUROC curve and calibration curves for the final model. The model had an AUROC curve for discrimination of patients with and without postoperative infection of 0.685. Internal calibration of the model was good, with a calibration in the large of − 0.04, a calibration slope of 1.06, and an observed/expected ratio of 0.97, though no bootstrapping was performed for internal validation, and external validation would be required to ensure predictive performance remains adequate.

**Table 6:** The final multivariable prediction model for predicting postoperative infections in patients undergoing surgery

Predictor	Coefficient	SE	Odds ratio
Chest tube tip culture^a^	0.661	0.414	1.94
Duration of postoperative drainage	0.098	0.043	1.10
Smoking status (former)	0.326	0.442	1.39
Smoking status (current)	− 1.363	0.765	0.26
Constant	− 2.140	0.498	

^a^Variables used in the full model included smoking status, chest tube tip culture, duration of postoperative drainage, diabetes, and type of surgery. The goal was to evaluate chest tube tip culture in combination with other variables, and as such it was kept in the reduced final model despite being non-significant

## Discussion

In our retrospective study we investigated if chest tube tip culture is useful for diagnosis of postoperative infections and found a sensitivity of 31%, a specificity of 83%, a PPV of 32% and a NPV of 83%. Subgroup analysis in patients with postoperative infection occurring *after* chest tube removal, resulted in a PPV of 18% and NPV of 92%, with similar sensitivity and specificity. Combining chest tube tip culture with additional variables in a multivariable model did not result in a meaningful improvement diagnostic value.

Culturing chest tube tips after thoracic drain removal has been routine practice in our hospital to check whether the patient might be at risk for a postoperative infection. However, this study shows that a positive chest tube tip culture is of poor diagnostic value for a postoperative infection after noninfectious lung surgery. Moreover, in the most frequent infectious postoperative complication, pneumonia, the majority of chest tube tips were cultured negative In addition, in the pneumonia cases with a positive chest tube tip culture, the cultured microorganisms on the tip and in the sputum causing the pneumonia, were not correlated.

A study previously performed by Yamauchi et al. [14] showed a lower sensitivity (23%), a higher specificity (98%) and a higher PPV (41%) compared to our study for predicting postoperative infections with chest tube tip culture. There are several explanations for these differences. First, the rate of positive chest tube tip cultures in our study was 19.5% compared to 3.8% reported in the study by Yamauchi et al. which could be due to the fact that Yamauchi et al. used sterile gloves and sterile gausses. This could have increased the number of positive tests in our study, resulting in a higher observed sensitivity and lower observed specificity. Second, the postoperative infection rate was 20% in our study compared to 5.8% in the study of Yamauchi et al., mostly owing to the difference in prevalence of postoperative pneumonia: 17% versus 2.5% in the study by Yamauchi et al. An explanation might be our definition for pneumonia of initiation of antibiotics for a suspected respiratory tract infection in contrast to the definition used by Yamauchi et al., which consisted of purulent sputum and pulmonary infiltrative changes on postoperative chest roentgenogram or computed tomography. Normally, higher prevalence of disease leads to an increase in PPV, which is not the case, most likely due to the counteracting effect of contamination resulting in a higher number of false-positive results.

Interestingly, in univariable analysis chest tube tip cultures were significantly associated with postoperative infection, SSI and empyema, but not with postoperative pneumonia. This finding is in contrast with the study of Yamauchi et al. [14], where a significant association with postoperative pneumonia was found. They substantiated this association using the theory of bacterial translocation across mesothelial cells suggested by Wilkosz et al. [21] However, none of our 9 patients with a postoperative pneumonia and a positive chest tube tip culture had the same organism isolated from their chest tube tip culture and their sputum culture. CNS were most often found in chest tube tip cultures from these patients and are highly unlikely as pathogens in pneumonia. Therefore, CNS are not reported in sputum culture result reports from most clinical microbiology laboratories. Nevertheless, these skin commensals are highly likely colonizing organisms of chest tubes and sometimes postoperative pleural empyema. Possibly the high concentration of antimicrobial peptides in postoperative pleural fluid studied by Hoetznecker et al. [22] provides additional protection against translocation of bacteria across mesothelial cells as well.

There are several limitations of this study. First, the retrospective study design resulted in different possible sources of bias. The clinicians were not blinded for the results of the chest tube tip culture, therefore a positive chest tube tip culture could have influenced the diagnosis of an SSI or the initiation of antibiotics for a suspected respiratory tract infection. This could lead to information bias, resulting in an overestimation of the diagnostic value. In addition, because we used routine hospital data, it is possible that diagnoses of postoperative infections were missed. Bailey et al. [23] found a wound infection rate in hospital records of 3% for patients who underwent hernia surgery, compared to 9% when additional information was obtained in the same patients. However, it is likely that the more severe and therefore clinically relevant SSIs were registered in the hospital records.

Secondly, in a minority of postoperative patients no chest tube tip culture was performed, partly because some patients spent a short postoperative period on the cardiothoracic ward or intensive care either. On these wards chest tubes were less frequently cultured compared to the pulmonary ward as a result of less strict adherence to protocol of routine cultures. These patients less frequently developed one or more postoperative infections compared to the group with chest tube tip cultures taken. In 9 out of 92 (10%) lung surgeries an infection was found without an available chest tube tip culture. Pneumonia, surgical site infection, and empyema were found in 8 (9%), 1 (1%) and 1 (1%) of these cases respectively. The higher rate of infections in the group of patients of whom a chest tube tip was cultured could result in a higher PPV and a lower NPV in that group.

Finally, both a strength and a limitation of the study is the single center design with a specific population in the UMCU. In our study, specific patient categories like patients with a history of cystic fibrosis and lung transplantation surgery were included. Patients with cystic fibrosis are often colonized with infectious pathogens which can inoculate the thoracic cavity during surgery and cause empyema [24, 25]. Impaired mucus secretion poses a risk for postoperative pneumonia as well. The increased susceptibly for infections in patients with a history of lung transplantation is mainly due to immunosuppression, decreased mucociliary clearance and decreased cough reflex [26]. This resulted in a significantly higher rate of postoperative infections in patients with a history of lung transplantation in our study. Our results are highly applicable to our patient groups. However, a multicenter design in various settings could have superior external validity.

## Conclusions

Our study shows that chest tube tip culture has poor accuracy in diagnosing postoperative infection in patients undergoing lung surgery for noninfectious disease. Therefore routine chest tube tip culture is not advisable and should be omitted to unburden the healthcare process and prevent low value care.

## Data Availability

The datasets used and/or analyzed during the current study are available from the corresponding author on reasonable request.

## Abbreviations

AUROC: Area under the receiver operator characteristic
BMI: Body mass index
CNS: Coagulase-negative staphylococci
NPV: Negative predictive value
PPV: Positive predictive value
SSI: Surgical site infection
UMCU: University Medical Center Utrecht
VATS: Video-assisted thoracoscopic surgery
